# Comparison of ophthalmic surgery rates in teprotumumab-treated vs. teprotumumab-untreated thyroid eye disease patients

**DOI:** 10.5935/0004-2749.2024-0319

**Published:** 2025-06-24

**Authors:** Taylor J. Linaburg, Tejus Pradeep, Brian Rinelli, Yuanyuan Chen, Yineng Chen, Gui-Shuang Ying, César A. Briceño, Madhura Tamhankar

**Affiliations:** 1 Department of Ophthalmology, Scheie Eye Institute, University of Pennsylvania, Philadelphia, PA, United States of America; 2 Department of Ophthalmology, Center for Preventive Ophthalmology and Biostatistics, University of Pennsylvania, Philadelphia, PA, United States of America

**Keywords:** Teprotumumab, Adrenal cortex hormone, Decompression, Graves ophthalmopathy, Strabismus

## Abstract

**Purpose:**

This study evaluated rates of thyroid eye disease-related eyelid surgeries, strabismus surgeries, and orbital decompressions in active thyroid eye disease patients treated with teprotumumab compared to those who were not.

**Methods:**

In this single-center longitudinal study, we compared patients with active thyroid eye disease evaluated from 02/01/2017 to 01/31/2020 (pre-teprotumumab era) with those seen from 02/01/2020 to 04/30/2023 (teprotumumab era). Patients from the pre-teprotumumab era who received corticosteroids and/or orbital radiation were compared with those in the teprotumumab era treated with teprotumumab, with or without corticosteroids and/or orbital radiation. The primary outcomes were rates of orbital decompressions, strabismus surgery, and eyelid surgery among patients with at least 6 months of follow-up. Orbital decompressions involving two or more walls were classified as severe.

**Results:**

Of 486 records reviewed, 106 patients had active thyroid eye disease. Among them, 33 were from the pre-teprotumumab era; 22 received corticosteroids and/or orbital radiation, and 11 received no treatment. Seventy three patients were from the teprotumumab era; 37 received teprotumumab (with or without corticosteroids and/or orbital radiation), 10 received corticosteroids and/or orbital radiation alone, and 26 received no treatment. Demographics were comparable between groups. Orbital decompression was performed in 11 of 44 eyes (25.0%) in the pre-teprotumumab era treated with corticosteroids and/or orbital radiation (8 one-wall, 3 ≥two-wall), compared to 3 of 74 eyes (4.1%) in the teprotumumab era treated with teprotumumab with or without corticosteroids and/or orbital radiation (all one-wall). The overall rate of orbital decompressions and the rate of ≥two-wall decompressions were significantly lower in the teprotumumab era (p=0.02 and p=0.0496, respectively). There was no significant difference in one-wall decompressions between era (p=0.07). Rates of strabismus surgeries (27.3% *vs.* 13.5%, p=0.19) and eyelid surgeries (22.7% *vs.* 21.6%, p=0.92) did not significantly differ between the era.

**Conclusions:**

In patients with active thyroid eye disease, treatment with teprotumumab was associated with a significantly lower rate and severity of orbital decompressions compared to treatment with corticosteroids and/or orbital radiation alone. However, the rates of strabismus and eyelid surgeries remained similar between groups.

## INTRODUCTION

Thyroid eye disease (TED) is an autoimmune disorder that affects the periorbital and orbital tissues. Its underlying mechanism involves overexpression and activation of the insulin-like growth factor 1 receptor (IGF-1R) in orbital fibroblasts, as well as B and T lymphocytes, leading to increased cytokine production and orbital inflammation^(1)^. Patients with TED may develop orbital fat and extraocular muscle hypertrophy, which can result in significant proptosis, diplopia, and, in rare cases, optic neuropathy and exposure keratopathy^(1^,^2)^.

Traditionally, treatment for active TED has included intravenous (IV), oral (PO), or periocular corticosteroids, orbital radiation, and/or surgery. Surgical interventions-orbital decompression (OrD), strabismus surgery (SS), and eyelid surgery (ES)-are usually reserved for the fibrotic phase of the disease, with the goal of reducing proptosis, diplopia, and addressing facial disfigurements. Many TED patients may ultimately require multiple surgeries, which contributes to the overall disease burden. Teprotumumab, an IGF1-R inhibitor approved by the US Food and Drug Administration in 2020 for the treatment of TED, interrupts the inflammatory process in retrobulbar tissues and represents a more targeted therapeutic approach^(3^-^5)^. Clinical studies have shown that teprotumumab is effective in improving proptosis, diplopia, and clinical activity score (CAS) in TED patients^(3^,^4^,^6)^. However, its effect on the frequency of TED-related surgeries has not yet been established. This study aims to evaluate the rate of TED-related surgeries-including SS, ES, and OrD-as well as the severity of OrD, in patients with active TED treated with teprotumumab compared to those who were not.

## METHODS

A single-center longitudinal cohort study was conducted, including unique patients diagnosed with TED based on ICD-10 codes, who were evaluated by study authors MT and CB in a joint TED clinic at the University of Pennsylvania between February 1, 2017, and April 30, 2023. Patients were divided into two cohorts: the pre-teprotumumab era (February 1, 2017, to January 31, 2020) and the teprotumumab era (February 1, 2020, to April 30, 2023). Inclusion criteria required a diagnosis of active TED, defined as an increase in proptosis of ≥2 mm from baseline and/or a CAS of ≥3 at presentation, along with a minimum of 6 months of follow-up after the final treatment. Patients were excluded if they had follow-up of less than 6 months, did not complete the full course of teprotumumab, received teprotumumab at a different institution, had undergone TED-related surgery before the study period or prior to starting medical therapy, died during the study period, or were enrolled in another study simultaneously. Collected data included demographics (age, gender, race, ethnicity), smoking status, proptosis measurements, CAS at presentation and post-treatment, and treatment details including IV and/or PO corticosteroids, orbital radiation, teprotumumab, and TED-related surgeries: OrD, SS, and ES. Steroid therapy was administered according to the EUGOGO guidelines for intermediate-dose IV methylprednisolone: 0.5 g once weekly for 6 weeks, followed by 0.25 g once weekly for another 6 weeks, for a total cumulative dose of 4.5 g. PO corticosteroids were used when IV administration was not feasible or based on patient preference^(7)^. Teprotumumab treatment followed the dosing regimen described in the clinical trial by Douglas et al., consisting of eight infusions-10 mg/kg for the initial dose, followed by 20 mg/kg for each of the subsequent seven infusions^(3)^. During the COVID-19-related pause on elective surgeries from March to May 2020 at the University of Pennsylvania (within the teprotumumab era), no patients required urgent OrD, all patients remained under follow-up, and elective surgeries were rescheduled for after May 2020. Clinic volume for TED evaluations remained stable, possibly due to continued demand for treatment in patients with active disease.

Practice patterns for medical and/or surgical management of TED patients by MT and CB in the joint TED clinic at the University of Pennsylvania remained consistent throughout the study period, as outlined below. In the pre-teprotumumab era, corticosteroids were initiated in cases of active TED without contraindications. Orbital radiation was considered for patients with contraindications to corticosteroids, those who did not respond to corticosteroids, or those who declined or were not suitable candidates for surgery. OrD surgery was primarily performed for cosmetic reasons and was typically reserved for the inactive phase of TED, except in urgent situations involving vision-threatening conditions such as compressive optic neuropathy (CON) or severe exposure keratopathy. In the teprotumumab era, teprotumumab was prescribed for active TED patients in the absence of contraindications. Teprotumumab could be used with or without prior corticosteroid therapy, although some insurance providers required corticosteroid treatment as a prerequisite for coverage. Corticosteroids were also used in patients with active TED in the absence of contraindications, who did not meet criteria for teprotumumab or preferred steroids for treatment. The indications for OrD remained the same as in the pre-teprotumumab era. The criteria for SS and ES were consistent across both eras: SS was performed for diplopia that impacted quality-of-life, while ES was indicated for eyelid malposition causing dryness, exposure keratopathy, or for cosmetic concerns that affected quality-of-life. In general, surgical intervention was deferred until at least 6 months after the final teprotumumab infusion, if administered, and only when the patient’s TED was deemed inactive and stable. An exception was made for cases of CON, where more urgent OrD was performed to preserve vision. These clinical decisions were considered within the broader context of patient-specific discussions about risks, benefits, and alternatives, emphasizing shared decision-making between physician and patient.

Active TED patients treated with corticosteroids and/or orbital radiation (S/OR) from February 1, 2017, to January 31, 2020 (pre-teprotumumab era), were compared to active TED patients treated with teprotumumab with or without S/OR from February 1, 2020, to April 30, 2023 (teprotumumab era). The primary outcomes were the rates of OrD, SS, and ES, with two-wall OrD classified as severe. Patient-level comparisons between the two eras were analyzed using two-sample t-tests for continuous variables and chi-squared or Fisher’s exact test for categorical variables. For eye-level outcomes such as OrD, generalized linear models were used, accounting for intereye correlation with generalized estimating equations. All statistical analyses were conducted using SAS version 9.4 (SAS Institute Inc., Cary, NC), with two-sided p-values <0.05 considered statistically significant. The study received Institutional Review Board approval from the University of Pennsylvania, complied with HIPAA regulations, and followed the principles of the Declaration of Helsinki.

## RESULTS

**Figure 1:**
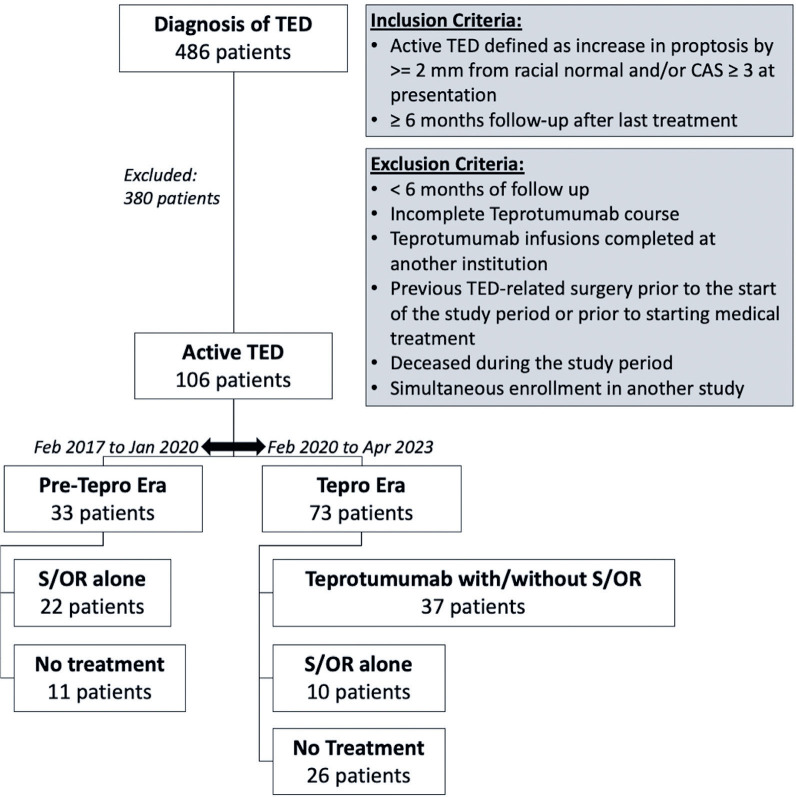
Breakdown of study cohort, including inclusion and exclusion criteria.

Of the 486 unique patients diagnosed with TED during the study period, 106 met the inclusion criteria for active TED (Figure 1). Among these, 33 patients were from the pre-teprotumumab era, with 22 of them receiving S/OR and 11 receiving no treatment. Of the 11 untreated patients in this group, eight were observed due to mildly active disease, two declined treatment, and one developed a rash following corticosteroid use and was subsequently monitored clinically. Seventy three patients were included in the teprotumumab era. Of these, 37 received teprotumumab with or without S/OR, 10 were treated with S/OR alone, and 26 received no treatment. Among the untreated patients in this era, 11 declined teprotumumab due to concerns about potential side effects, 10 had mildly active disease and opted for observation, 1 was denied insurance coverage for teprotumumab, and 4 had medical conditions such as a history of irritable bowel disease, prediabetes or diabetes mellitus, atrial fibrillation, or uncontrolled hyperthyroidism that contraindicated teprotumumab use. The 10 patients who received only S/OR did so due to patient preference, insurance denial, or contraindications to teprotumumab. None of the patients treated with teprotumumab reported adverse effects that required discontinuation of therapy or additional medications to manage symptoms. Demographic characteristics-including age, gender, smoking status, average duration of follow-up, average CAS, and average proptosis at presentation-were similar between the pre-teprotumumab and teprotumumab era groups (Table 1).

**Table 1:** Demographics and TED metrics for pre-teprotumumab era *vs.* teprotumumab era cohorts

	Pre-teprotumumab era (n=33)	Teprotumumabera (n=73)
Mean (SD) age (years)	65 (15)	56 (14)
Gender (# (%))		
Male	10 (30)	23 (31)
Female	23 (70)	50 (69)
Race (# (%))		
White	21 (64)	41 (56)
Black	6 (18)	15 (20)
Asian	1 (3)	7 (10)
Others	5 (15)	10 (14)
Smoking status (# (%))		
Nonsmoker	20 (61)	41 (56)
Current or former	11 (33)	29 (40)
Unknown	2 (6)	3 (4)
Mean (SD) of follow-up (months)	30.5 (22.1)	21.7 (14.1)
Mean (SD) of CAS at presentation	3.9 (1.1)	4.2 (1.1)
Mean (SD) of proptosis at presentation (mm)	21.8 (4.7)^^*^^	21.8 (3.3)^#^

TED= thyroid eye disease; CAS= clinical activity score; SD= standard deviation.

^^*^^n=31, 62 eyes;

# n=72, 144 eyes.

TED= thyroid eye disease; CAS= clinical activity score; S/OR, corticosteroids and/or orbital radiation.

There was no statistically significant difference in the percentage of patients who were monitored without medical treatment between the two cohorts (p=0.82, Table 2). While the percent of patients who received corticosteroids was lesser in the teprotumumab era compared to the pre-teprotumumab era, this difference was not statistically significant (p=0.12, Table 2). The proportion of patients who received orbital radiation in the teprotumumab era was significantly lower than in the pre-teprotumumab era (p<0.001, Table 2).

**Table 2:** Comparison of medical treatments between pre-teprotumumab era *and* teprotumumab era

	Pre-teprotumumab era (n=33)	Teprotumumab era (n=73)	p-value
No medical treatment, n (%)	11 (33.3)	26 (35.6)	0.82^*^
Corticosteroids, n (%)	18 (54.5)	28 (38.4)	0.12^*^
Orbital radiation, n (%)	9 (27.3)	1 (1.4)	<0.001#
Teprotumumab, n (%)	0 (0.0)	37 (50.7)	<0.001#

* p-value from chi-squared or

# Fisher’s exact test.

In the pre-teprotumumab era, 11 out of 44 eyes (25.0%) underwent OrD; 8 had one-wall OrD, and 3 had ≥two-wall OrD. In contrast, only 3 out of 74 eyes (4.1%) in the teprotumumab era underwent OrD, all with one-wall OrD. The rate of OrD and ≥two-wall OrD was significantly lower in the teprotumumab era compared to the pre-teprotumumab era (p=0.02 and p=0.0496 respectively), while the difference in one-wall OrD between the eras was not statistically significant (p=0.07). The treatments received prior to surgery for each group are detailed in Table 3. The mean follow-up for all patients who underwent OrD in both eras was 25 months, and none experienced regression or reactivation that impaired cosmetic appearance, requiring repeat interventions, either medically or surgically, during follow-up. The rates of SS and ES did not differ significantly between the pre-teprotumumab and teprotumumab eras (27.3% *vs.* 13.5%, p=0.19; 22.7% *vs.* 21.6%, p=0.92, respectively) (Table 3).

**Table 3:** Comparison of TED-related surgeries between pre-teprotumumab era (S/OR alone) *and* teprotumumab era (teprotumumab +/- S/OR alone)

	Pre-teprotumumab era: S/OR alone (n=22 patients, 44 eyes)	Teprotumumab era: teprotumumab +/ S/OR (n=37 patients, 74 eyes)	p-value
OrD, number of eyes (%)	11 (25.0)	3 (4.1)	0.02
One-wall, number of eyes (%)	8 (18.2%) Steroids alone (n=4)^*^ steroids + orbital radiation (n=4)^†^	3 (4.1%) Steroids + tepro (n=2)^‡^ Tepro alone (n=1)	0.07
≥two-wall, number of eyes (%)	3 (6.8) steroids alone (n=3)^‡^	0 (0.0)	0.0496
Strabismus surgery, number of patients (%)	6 (27.3) Steroids alone (n=4) Orbital radiation alone (n = 1) steroids + orbital radiation (n=1)	5 (13.5) Steroids + tepro (n=3) Tepro alone (n=2)	0.19
ES, number of patients (%)	5 (22.7) Steroids alone (n=4) Orbital radiation alone (n=1)	8 (21.6) Steroids + tepro (n=5) Tepro alone (n=3)	0.92

TED= thyroid eye disease; S= corticosteroids; CON= compressive optic neuropathy; S/OR= corticosteroids and/or orbital radiation.

^^*^^3 eyes with CON;

† 4 eyes with CON;

‡ 2 eyes with CON.

In the pre-teprotumumab era, 11 eyes had CON, 9 of which underwent OrD due to failure of corticosteroids (5 eyes) or failure of combination treatment with corticosteroids and orbital radiation (4 eyes). Two eyes with CON did not require OrD as they responded well to corticosteroids alone. Seven of eight (87.5%) one-wall OrDs were in eyes with CON, and two of three (66.7%) ≥two-wall OrD were in eyes with CON (Table 3). In the teprotumumab era, seven eyes had CON, with two undergoing one-wall OrD due to failure of combination treatment with corticosteroids and teprotumumab. The remaining five eyes did not require OrD due to a good response to corticosteroids (two eyes), teprotumumab (two eyes), or a combination of both treatments (one eye). Two of the three one-wall OrDs (66.7%) were in eyes with CON, and no eyes required ≥two-wall OrD in the teprotumumab era (Table 3).

## DISCUSSION

Our data indicates that teprotumumab treatment significantly reduced the rate and severity of OrD surgeries in active TED patients compared to those treated with S/OR alone. However, it did not affect the frequency of SS and ES. Among patients requiring OrD, the majority had minimal or no response to S/OR or teprotumumab, which led to the decision to pursue surgical management. A higher proportion of patients with CON in the teprotumumab era were able to avoid OrD when treated with teprotumumab, either alone or with corticosteroids, compared to the pre-teprotumumab era when they were treated with S/OR alone.

Teprotumumab has been shown to reduce proptosis, as demonstrated in two clinical trials^(3^,^4)^. Additionally, the OPTIC study reported that teprotumumab reduces orbital soft tissue volume, with a subset of patients showing a decrease in orbital fat and extraocular muscle volumes through imaging before and after treatment^(8)^. These anatomical changes were largely unaffected by corticosteroids and other immunosuppressive agents previously reported in the literature^(8)^. Consistent with this, a recent study examining proptosis regression over time after teprotumumab treatment found that most TED patients maintained a statistically significant reduction in proptosis compared to pretreatment levels, indicating a long-term benefit, although the reduction may be less pronounced than immediately after treatment^(6)^. Together with our findings, this suggests that teprotumumab’s ability to sustain proptosis improvement may reduce the need for fewer and/or less severe OrD surgeries.

A recent study by Ugrader and colleagues found a reduction in orbital fat and extraocular muscle volumes on radiographic imaging in patients with chronic TED treated with teprotumumab, similar to findings in patients with active TED^(9)^. This suggests that teprotumumab may partially reverse the remodeling of orbital tissues in TED, regardless of disease activity as measured by CAS. Although our study did not focus on patients with chronic, inactive TED, future research on teprotumumab in both active and chronic/inactive TED patients may provide further insights into its effects on orbital fat and extraocular muscle volumes, proptosis, and potentially a reduced need for OrD in both patient groups.

There was no difference in the rate of ES between patients treated with S/OR in the pre-teprotumumab era and those treated with teprotumumab with or without S/OR in the teprotumumab era. Upper eyelid retraction is independent of globe position and proptosis, while lower eyelid retraction is more closely associated with globe position. Therefore, while upper eyelid retraction often persists after proptosis reduction, either medically or surgically, lower eyelid retraction may improve in patients with minimal eyelid laxity^(10^,^11)^. Consistent with this, all five pre-teprotumumab era patients treated with S/OR and all eight teprotumumab era patients treated with teprotumumab with or without S/OR who required ES underwent upper eyelid procedures, including blepharotomies, blepharoplasties, or lateral tarsorrhaphies.

There was no difference in the rate of SS between the two study groups, and notably, no patients in either the pre-teprotumumab or teprotumumab era cohorts underwent SS following initial OrD in this study. This is important to note, as OrD surgery itself can sometimes induce intolerable diplopia, which could lead to SS, not because of primary TED but due to the effects of the OrD surgery. While Smith et al. reported that 68% of teprotumumab-treated patients experienced subjective improvement in diplopia compared to 26% of controls, and the OPTIC study found that 68% of teprotumumab--treated patients had a ≥1 Gorman grade reduction in diplopia, neither study addressed whether these patients still required SS to achieve their vision goals^(3)^. Therefore, while teprotumumab may improve proptosis, CAS, and diplopia in active TED patients, some may still need SS to further improve their diplopia and overall quality-of-life.

The percentage of patients receiving corticosteroids as part of their treatment regimen was similar in both cohorts. This is likely due to delays in teprotumumab authorization, the need for treatment during the waiting period before teprotumumab infusions, physician unfamiliarity with the new medication, and the disruption of teprotumumab production during the COVID-19 pandemic from December 2020 to February 2021^(12)^. The use of corticosteroids while waiting teprotumumab approval may continue until the approval process for teprotumumab is expedited.

Fewer patients in the teprotumumab era received orbital radiation compared to the pre-teprotumumab era. The effectiveness of orbital radiation for treating active TED has been debated for years, and as a result, there has been a shift from using orbital radiation to adopting targeted therapies like teprotumumab among treating physicians.

Our study has several limitations. It is a retrospective, single-institution study with a small cohort of patients. Although we reviewed 486 TED patient records, 78% were excluded from our analysis (see Methods and Figure 1). The strict inclusion and exclusion criteria were necessary to establish two well-matched cohorts with comparable TED activity for meaningful comparisons. Notably, the teprotumumab era cohort had a disproportionately larger number of patients. This is likely due to several factors, including the following: (1) The University of Pennsylvania TED clinic is part of a large academic institution, which may introduce referral bias. (2) Increased awareness of teprotumumab has led to more TED patients to seek evaluation at larger academic institutions offering the medication, which may further contribute to referral bias; (3) There is likely a change in attitude towards surgery from both the physician and patient perspective with the advent of teprotumumab; reduced patient consideration of surgery/OrD would subsequently affect rate of surgeries in the teprotumumab era and not in the pre-teprotumumab era.

In the USA, the commercial cost of teprotumumab ranges from $431,000 to $981,000, with a median cost of $823,000^(13)^. Additionally, in a study by Shah et al. evaluating treatment costs of medical options for TED, treatment cost/ΔGO-QoL (change in Graves’ orbitopathy quality-of-life score) of teprotumumab was found to be nearly 20 times that of rituximab and around 50 times that of IV methylprednisolone^(13)^. Due to its high cost, obtaining insurance approval for teprotumumab is challenging. Comparing this medication cost to OrD surgery is difficult because surgery costs vary based on the location, the extent of decompression required, and the level of insurance coverage. Additionally, the risks and outcomes of both teprotumumab and surgery must be considered. For teprotumumab, these include potential sensorineural hearing loss and gastrointestinal issues, while surgery carries risks such as iatrogenic diplopia, damage to the globe or extraocular muscles (EOM), vision loss, general anesthesia complications, long recovery times, and the possibly of requiring further procedures for OrD. The decision to pursue teprotumumab versus OrD is made on an individual basis, and the best option may not always be covered by insurance or affordable for the patient.

Our data show that teprotumumab treatment significantly reduced the rate and severity of OrD surgeries for active TED patients compared to treatment with S/OR alone. However, it did not reduce the rate of SS or ES compared to treatment with S/OR alone. Teprotumumab is rapidly becoming the first-line treatment for active TED in the USA. However, there are still gaps in the literature concerning its cost-effectiveness, potential side effects, long-term safety, and the benefits of teprotumumab in reducing the frequency of TED-related surgeries. Future studies are needed to address these issues and guide clinical decisions regarding the long-term use of teprotumumab for patients with active, chronic, or reactivated TED.
